# Diabetic Patients' Experiences in Primary Healthcare Centers in the Al-Ahsa Region, 2025

**DOI:** 10.7759/cureus.90843

**Published:** 2025-08-23

**Authors:** Ali A Bu-khamseen, Abdullah AlQuwidhi, Malak Alhaddad, Mohammad Alalawi, Ali A Alkuwaiti, Tamim Alsultan

**Affiliations:** 1 Family Medicine, Primary Health Care, Al-Ahsa, SAU

**Keywords:** al-ahsa, diabetic patients, patient experience, primary healthcare, team-based care

## Abstract

Background

As part of Saudi Arabia's Vision 2030 healthcare transformation, assessing patient experience has become essential to improving the quality of care, particularly in managing chronic diseases such as diabetes. Despite increasing global interest, few studies have explored patient experiences in primary healthcare centers (PHCs) in the Al-Ahsa region.

Objective

This study aimed to evaluate the experience of diabetic patients attending PHCs in Al-Ahsa, focusing on communication, accessibility, coordination of care, and clinical support services.

Methods

This cross-sectional study was conducted between January and March 2025 across 66 PHCs in Al-Ahsa. A total of 392 patients with diabetes were selected through simple random sampling. Data were collected via validated telephone interviews and analyzed using descriptive statistics, t-tests, one-way analysis of variance (ANOVA), and linear regression.

Results

The overall patient experience score was rated “excellent” (mean = 2.7/3.0). Domains, such as respect for privacy, consultation time, and nurse attentiveness, had the highest scores. Male sex, higher educational attainment, and routine follow-up with a care team were significantly associated with higher experience scores (p < 0.05). Patients with type 2 diabetes and uncontrolled diabetes also reported a better experience. Regression analysis confirmed that educational level was a significant predictor of experience (B = 0.030-0.032, p = 0.001).

Conclusion

The findings demonstrate excellent experiences among diabetic patients in Al-Ahsa PHCs, highlighting the importance of education, health literacy, and continuity of care in enhancing patient-centered outcomes. Tailored interventions that focus on demographic subgroups may further improve patient engagement and service quality.

## Introduction

Patient experience is defined as a wide range of interactions between the patient and the healthcare system, encompassing the care of staff in the hospital, plans for their health, and healthcare facilities. Patient experience is key to quality care, involving communication, appointment access, coordination, and self-management [1]. A Spanish study showed that measuring patient experience helps guide patient-centered care for chronic diseases [2]. Two systematic reviews demonstrated that patients have a common link between healthcare, healthcare staff and treatment; therefore, they are reliable sources of information for patient safety [3,4]. It can also be used to develop plans to improve health care [3]. A recent study has demonstrated multiple aspects of patient experience. Relational experience focuses on the relationship between the doctor and the patient and has four domains. The first domain, compassion, measures how patients are treated, including ensuring privacy, showing empathy, and providing emotional support. The second domain is communication, which measures communication between physicians and patients, including providing information and education, obtaining consent, and enrolling the patient in the plan of treatment. Third domain: timelines that measure the different kinds of delay, time spent waiting, access to facilities, and cancelation. The fourth domain is reliability, which measures the patient’s perception about the management of the unit, including safety, availability of information such as lab results, efficiency, and dependability. The satisfaction of the results focuses on the direct benefit to the patient after the consultation and has four domains. The first domain is consultation, which includes face-to-face and virtual consultations. The second domain includes treatment with different kinds of care, therapy, prescribed, or adjusted medications. The third domain is practical help, such as arrangements. Fourth domain: plans that include future plans, extra tests, or referrals to specialists. Service integration is linked to how the patient experienced care and has four domains. The first domain, communication, measures the communication between services. The second domain is service knowledge, which measures how the patient perceives the knowledge of the staff about other services. The third domain, repetition, measures whether the patient needs to repeat his story to every staff member. The fourth domain, collaboration, measures whether different services work as a team [5]. Team-based health care is defined as the delivery of healthcare to patients by at least two healthcare providers who work together with patients and their caregivers to achieve shared goals with the best quality of care [6]. It embarked on reforming its health sector as part of a wider agenda for transforming all government sectors, as envisioned in Vision 2030 and the National Transformation Program 2020. One goal is to improve health and extend life expectancy to 80 years by 2030, aligning with Vision 2030. Another goal is to enhance healthcare quality, consistency, and accountability for safe, timely, and patient-centered care. The third one is to improve value by containing costs, improving outcomes, controlling public healthcare expenditure, and guiding new investments [7].

An efficient healthcare system must include primary healthcare center (PHC). Saudi Arabia prioritizes PHCs in reforms to tackle the growing burden of non-communicable diseases [8]. PHC is defined as an approach to health for the entire society that targets the delivery of the best level of care. In addition, it focuses on society's needs and continuity of care in terms of prevention, promotion of health, prevention and management of diseases, rehabilitation and visibility to everyone [9]. 

Diabetes mellitus (DM) is a chronic and progressive, medically complex condition characterized by abnormally high blood glucose levels. Type 2 diabetes mellitus (T2DM) and type 1 diabetes mellitus (T1DM) are the most common types of diabetes [10]. The global prevalence of DM is increasing, which may cause signs and symptoms as well as serious long-term sequelae. DM affects almost every organ of the body, primarily because of metabolic disturbances caused by hyperglycemia, especially if the control of diabetes is poor over extended periods. Complications such as blindness, renal impairment, and amputation [11,12]. Moreover, cardiovascular disease kills three-quarters of people with T2DM [13]. Treatment has focused on drug interventions to manage hyperglycemic levels and decrease cardiovascular disease risk factors, such as lipid profile and blood pressure, to lower the likelihood of cardiovascular events over time [14]. DM has emerged as a major global public health issue, particularly in developing countries [15]. Diabetes is not only a global health issue because of its effect on mortality, morbidity, and quality of life, but it is also a major problem for national economies [16]. This causes a rising financial burden on the healthcare system, specifically related to hospital inpatient treatment [17]. The cost of diabetes worldwide is $1.31 trillion, or 1·8% of the global gross domestic product (GDP) [18]. In the United States, the anticipated overall cost of diabetes was $327 billion, comprising $237 billion for direct medical expenses and $90 billion for reduced productivity. Medical expenses for people with DM are typically 2.3 times higher than they would be in the absence of the disease [19]. Furthermore, diabetes can substantially affect individuals through loss of productivity, premature mortality, and mental disorders [20,21]. Among diabetic patients, 30% have mental illnesses such as schizophrenia, depression, delirium, and drug abuse (e.g., smoking tobacco) [21]. People with DM are two to three times more likely to experience depression [22]. According to the most recent International Diabetes Federation (IDF) report, by 2045, there would be 783.2 million individuals (12.2% of the global population) with diabetes, up from 536.6 million adults (10.5%) in 2021 [13]. It will continue to rise to the point that by 2050, between one in five and one in three persons will have diabetes [23]. The Kingdom of Saudi Arabia (KSA) is not excluded from this global epidemic, and is the most challenging health issue that the country is currently confronting [24]. In 2015, Saudi Arabia was among the top 10 countries in the world for the prevalence of diabetes [25]. A report from the Saudi Arabian Ministry of Health, published in 1992, estimated that 0.9 million persons had diabetes; however, by 2010, that number had increased to 2.5 million, indicating a 2.7-fold increase in incidence rates in less than 20 years [26]. According to the IDF, the overall prevalence of diabetes in Saudi Arabia in 2021 is 17.7%, with a total cases of 4,274,100 [27].

Literature on diabetes has focused on the quality of care and its related indicators and measurements, as it has become an important focus of the healthcare system and policy [28]. One of the important factors in assessing the impact of a behavioral change program is the evaluation of patient experience, as it is their perception of the program components that influence health behavioral changes [29]. To enhance the eventual and overall management of diabetes, increasing efforts are being made to measure patients' experience of the effects of the disease and its treatment [30]. It was proven that when physicians and healthcare workers listen to their patients' narratives regarding their clinical encounters and experiences, they will be able to improve according to the defect in the service. In a study conducted in London, the Diabetes Treatment Satisfaction Questionnaire (DTSQ) was used to assess outcomes, especially psychological outcomes, in diabetes treatment plans, and it turned out that it is influenced by some interventions, such as the route of medication from oral agents to injectable therapy and switching between types of insulin [30]. A study conducted in the Department of Family Medicine and PHC, King Abdulaziz Medical City, studied the association between overall patient satisfaction and diabetes outcomes and found no statistical correlation between HbA1C and satisfaction, unlike other studies, which proved this association [28].

This study aims to evaluate the patient experience of individuals with diabetes attending primary healthcare centers in Al-Ahsa, Saudi Arabia. It also seeks to identify various factors that may influence patient experience. We hypothesize that measuring patient experience will enhance value-based care for patients with diabetes.

## Materials and methods

Study area and period

This study was carried out in PHCs, including four clusters (east, west, north, and south) covering 66 PHCs in Al-Ahsa, Eastern Province, Kingdom of Saudi Arabia. The study period was between January 2025 and March 2025.

Study design and population 

 It is a descriptive cross-sectional study. The study population consisted of all diabetic patients registered in primary healthcare centers (PHCs), estimated to be approximately 55,000 individuals. The inclusion criteria included patients with type 1 and type 2 diabetes who were registered in PHCs within the Al-Ahsa region. Patients diagnosed with maturity-onset diabetes of the young (MODY), latent autoimmune diabetes in adults (LADA), and gestational diabetes were excluded from the study.

Sample size

After reviewing the number of registered patients with diabetes in the healthcare electronic system (HIS), there were 55,000 files. The sample size was calculated with a margin of error of 5% and a 95% confidence interval. The estimated sample size was 392 patients. 

Sampling technique

Simple random sampling that included all registered diabetic patients in the primary healthcare electronic system (HIS).

Data collection tool and technique

Data were collected through structured telephone interviews conducted by a trained data collector using the office phone system in the family medicine clinic. Each participant received a phone call during which the data collector introduced themselves, provided a brief overview of the study, and obtained verbal consent before starting the questionnaire, which was generated by the research and validated through a pilot study in which Cronbach's alpha value was 0.868, indicating good internal consistency and reliability for research purposes.

Questionnaire components

This comprehensive questionnaire gathered key clinical and experiential data from patients. It began by recording the most recent A1C level, sourced directly from the medical record rather than patient self-report. Demographic data collected included age, gender, educational level, marital status, employment status, the type of usual consultation for diabetes follow-up (with or without appointment), and whether the patient consistently saw the same medical team. Regarding diabetes history, information was captured on the type of diabetes (T1DM, T2DM, or unknown), management type (oral medications, injectable therapy, both, or lifestyle modification only), and method of diagnosis (symptomatic presentation, screening, or incidentally). Participants were also asked if they were aware of their latest A1C result. The presence of comorbidities (hypertension, dyslipidemia, obesity, or none) and diabetes-related complications (diabetic foot, retinopathy, nephropathy, neuropathy, or none) was also documented. To assess patient experience, the survey evaluated eight domains: access to care, moving through the healthcare system, interactions with the nurse assistant, interactions with the physician, investigations, pharmacy services, and personnel issues. This section employed a three-point Likert scale with response options "Excellent," "Average," and "Poor". Interpretation of the mean scores for these domains was guided by defined categories: scores from 1.00 to 1.60 indicated a "Poor" (very low/unsatisfactory) level; scores from 1.67 to 2.30 indicated an "Average" (moderate/acceptable) level; and scores from 2.31 to 3.00 indicated an "Excellent" (high/outstanding) level (see Appendix, Tables 7, 8).

Statistical analysis

All statistical analyses were performed using SPSS version 26 (IBM Corp., Armonk, New York, USA). The significance level was set at α = 0.05 for all inferential tests. Descriptive statistics, including means, standard deviations, frequencies, and percentages, were used to summarize the participant demographics and survey responses. Cronbach's alpha was used to evaluate the internal consistency of the patient experience scale, and it was 0.868. According to conventional thresholds in psychometric testing, a Cronbach's alpha value above 0.8 is considered to indicate good internal consistency. The relationships between continuous variables, such as age and experience scores, were examined using Pearson's correlation coefficient.

To analyze differences in patient experience scores, several statistical methods were employed. Independent samples t-tests were used to compare mean scores between two distinct groups, such as males versus females, patients with controlled versus uncontrolled diabetes, or different follow-up statuses. When comparing means across more than two groups (e.g., marital status categories, occupation types, educational levels, or diabetes types), one-way analysis of variance (ANOVA) was applied. For any statistically significant ANOVA results, Duncan's post hoc test was subsequently performed to identify homogeneous subgroups sharing similar mean scores. To identify predictors of patient experience, linear regression analyses were conducted. These regression models incorporated key sociodemographic variables, including age, gender, marital status, occupation, and educational level. The regression outputs comprising R-squared values, p-values, and regression coefficients were examined to determine the strength, direction, and significance of each predictor's association with experience scores. Finally, the three-point Likert scale responses ("Excellent," "Average," and "Poor") were summarized using mean scores, which were categorized into three interpretive ranges: Poor (1.00-1.60), Average (1.67-2.30), and Excellent (2.31-3.00) for clear reporting and analysis.

*Description of the Experience Score Variables* 

Two composite experience scores were calculated to assess patient experience. The first, termed the average score (with Q3), included responses from all survey variables, including question 3 (Q3). The second composite score excluded Q3 to evaluate whether this specific question influenced the overall results. These are listed in Table 1.

**Table 1:** Average scoring. Note to question 2: "No" → Score 1: The patient knows the medical team but does not follow up with them. "Yes" → Score 1: The patient knows the medical team and does follow up with them. "I don't know my medical team" → Score 0: The patient is not aware of who their medical team is.

Question	Choice	Score
A1: What method do you usually use for medical consultation related to diabetes?	Appointment booked	2
	Call 937	2
	My Health app	2
	Without appointment	1
A2: Do you follow up with your medical team every time?	I don't know my medical team	0
	Yes	1
	No	0
A3: What type of diabetes do you have?	Type 2	1
	Type 1	1
	I don't know	0
A4: What is the method of diagnosis?	Incidentally	0
	I had symptoms	1
	Early screening programs	2
A5: What is your current treatment?	Injections and pills	2
	Pills	1
	Injections	2
	Only a healthy lifestyle	3
A6: Do you know the result of the A1C test?	No	0
	Not sure	1
	Yes	2
Q1-Q19: Patient experience	Excellent	3
	Average	2
	Poor	1

Ethical considerations

Ethical clearance obtained from the Prince Saud Bin Jalawi Hospital Ethics Committee (IRB No. H-05-HS-135). IRB Log No: 19-EP-2024. Patient data confidentiality maintained and used for research. There is no conflict of interest. This study is self-funded.

## Results

The mean age of participants was 54.16 years (SD = 12.85), with a range of 22 to 91 years. The mean HbA1c level was 7.9% (standard deviation (SD) = 1.79), ranging from 5.0% to 13.8%. Table 2 provides detailed biographical information for the 392 participants.

**Table 2:** Sociodemographic and clinical characteristics of the study participants.

Variable	Group	Count (N)	Percent (%)
Gender	Female	191	48.7
	Male	201	51.3
Marital status	Married	310	79.1
	Separated	11	2.8
	Single	24	6.1
	Widowed	47	12.0
Occupation	Freelance	27	6.9
	Government sector	74	18.9
	Not working	177	45.2
	Private sector	43	11.0
	Retired	71	18.1
Educational level	Bachelor	100	25.5
	Diploma	31	7.9
	Elementary	65	16.6
	High School	97	24.7
	Illiterate	55	14.0
	Secondary School	44	11.2
Do you have health insurance?	I had it previously	11	2.8
	No	305	77.8
	Yes	76	19.4
What method do you usually use for medical consultation related to diabetes?	Appointment booked	85	21.7
	Call 937	27	6.9
	My Health app	212	54.1
	Without appointment	68	17.3
Do you follow up with your medical team every time?	No	226	57.7
	Yes	166	42.3
What type of diabetes do you have?	I don't know	125	31.9
	Type 1	28	7.1
	Type 2	239	61.0
What is the method of diagnosis?	Early screening programs	189	48.2
	I had symptoms	156	39.8
	Incidentally	47	12.0
What is your current treatment?	Injections	35	8.9
	Injections and pills	55	14.0
	Only a healthy lifestyle	16	4.1
	Pills	286	73.0
Do you know the result of the A1C test?	No	74	18.9
	Not sure	54	13.8
	Yes	264	67.3
Do you have any of the following complications?	I do not have any of the mentioned complications	271	60.49
	Retinopathy	60	13.39
	Peripheral neuropathy	57	12.72
	Kidney failure	40	8.93
	Diabetic foot	20	4.46
Do you have any conditions associated with diabetes?	I do not have any of the mentioned diseases	174	32.95
	High blood pressure	167	31.63
	High cholesterol	105	19.89
	Obesity	82	15.53

Table 3 shows the count and percentage values for each question, along with mean values and the direction. Overall, the total shows that across all items, 5608 responses (75.3%) were marked Excellent, 1461 (19.62%) Average, and 379 (5.09%) Poor, with an overall mean experience score of 2.7, which falls in the "Excellent" range according to the scale's defined interpretation. Survey items Q1-Q19, listed in Table 3.

**Table 3:** Patient experience rating across different stages of the clinical visit.

Category	Question	Excellent	Average	Poor	Mean	Direction
		N(%)	N(%)	N(%)		
Registration	Ease of appointment booking	299 (76.28%)	71 (18.11%)	22 (5.61%)	2.71	Excellent
Registration	Availability of appointments	224 (57.14%)	119 (30.36%)	49 (12.5%)	2.45	Excellent
Registration	Ease of registration procedures at reception	330 (84.18%)	55 (14.03%)	7 (1.79%)	2.82	Excellent
During your visit	Clarity of the navigation plan after registration	325 (82.91%)	56 (14.29%)	11 (2.81%)	2.8	Excellent
During your visit	Waiting time before entering the vital signs area	267 (68.11%)	116 (29.59%)	9 (2.3%)	2.66	Excellent
During your visit	Waiting time before seeing the doctor	204 (52.04%)	161 (41.07%)	27 (6.89%)	2.45	Excellent
During your visit	Time spent during the consultation	343 (87.5%)	44 (11.22%)	5 (1.28%)	2.86	Excellent
Doctor	The explanation your doctor provides about your problem or health condition	324 (82.65%)	58 (14.8%)	10 (2.55%)	2.8	Excellent
Doctor	The concern your doctor shows regarding your questions or concerns	347 (88.52%)	42 (10.71%)	3 (0.77%)	2.88	Excellent
Doctor	Your doctor's effort to involve you in decisions about your treatment	313 (79.85%)	59 (15.05%)	20 (5.1%)	2.75	Excellent
Doctor	Doctor's discussion of treatment options (choices)	282 (71.94%)	78 (19.9%)	32 (8.16%)	2.64	Excellent
Nursing	The extent to which the nurse listened to you	331 (84.44%)	60 (15.31%)	1 (0.26%)	2.84	Excellent
Nursing	The concern the nurse showed for your health issue	323 (82.4%)	64 (16.33%)	5 (1.28%)	2.81	Excellent
Laboratory	Waiting time before the blood sample is taken	233 (59.44%)	141 (35.97%)	18 (4.59%)	2.55	Excellent
Laboratory	Time required for the results to appear	290 (73.98%)	80 (20.41%)	22 (5.61%)	2.68	Excellent
Pharmacy	Availability of prescribed medications	239 (60.97%)	127 (32.4%)	26 (6.63%)	2.54	Excellent
Follow-up	You are given a follow-up appointment after each visit	243 (61.99%)	60 (15.31%)	89 (22.7%)	2.39	Excellent
Personal matters	Respect for your privacy	368 (93.88%)	22 (5.61%)	2 (0.51%)	2.93	Excellent
Personal matters	Extent to which the medical staff ensured your safety (hand sanitizing)	323 (82.4%)	48 (12.24%)	21 (5.36%)	2.77	Excellent
#	Total	5608(75.3%)	1461(19.62%)	379(5.09%)	2.7	Excellent

Table 4 shows that age was not significantly correlated with patient experience scores (Pearson r ≈ -0.07, p > 0.15). Independent-sample t-tests revealed that male patients reported slightly higher scores than females (p < 0.05). One-way ANOVA showed experience differed by marital status, with married patients rating highest and separated patients lowest (p < 0.05). Occupational group also mattered (p = 0.001): government employees scored best, while freelance workers scored worst. Education level followed the same pattern (p = 0.001), with bachelor-plus respondents topping the ratings. Diabetes type affected scores too (p = 0.001), as type 2 patients outperformed both type 1 and "I don't know" groups. Uncontrolled diabetics reported modestly better experiences than controlled patients (p = 0.001), and those who followed up with their medical team rated their care higher than those who did not (p = 0.001).

**Table 4:** Associations between demographic and clinical factors and patient experience scores.

Question number	Variable pair	Average score (with Q3)	P-value	Average score (without Q3)	P-value
Q2: Age and experience	Correlation coefficient	-0.073	0.15	-0.06	0.238
Q3: Gender	Female	2.24±0.24	0.026	2.30±0.25	0.02
	Male	2.29±0.23		2.36±0.23	
Q4: Marital status	Single	2.23±0.27	0.026	2.29±0.27	0.032
	Married	2.28±0.22		2.34±0.22	
	Widowed	2.21±0.25		2.27±0.25	
	Separated	2.11±0.42		2.18±0.44	
Q5: Occupational group	Not working	2.22±0.24	0.001	2.29±0.25	0.001
	Freelance	2.14±0.31		2.21±0.31	
	Government sector	2.37±0.17		2.43±0.17	
	Private sector	2.33±0.19		2.39±0.20	
	Retired	2.25±0.22		2.32±0.23	
Q6: Educational level	Illiterate	2.21±0.25	0.001	2.28±0.25	0.001
	Elementary	2.22±0.26		2.29±0.26	
	Secondary	2.21±0.31		2.27±0.31	
	High school	2.23±0.21		2.28±0.21	
	Diploma	2.28±0.21		2.34±0.22	
	Bachelor+	2.37±0.17		2.43±0.17	
Q8: Type of diabetes	I don't know			2.24±0.26	0.001
	Type 1			2.27±0.27	
	Type 2			2.38±0.21	
Q9: Controlled vs uncontrolled	Controlled	2.22±0.25	0.001	2.28±0.26	0.001
	Uncontrolled	2.30±0.211		2.37±0.215	
Q10: Follow-up with the medical team	No/I don't know			2.27±0.25	0.001
	Yes			2.42±0.18	

Table 5 shows that the model including Q3 shows a slightly higher R-square value (0.056 vs. 0.046) and F-statistic (22.938 vs. 18.987), indicating a modest improvement in model fit when Q3 is included. Both models are statistically significant. Adding Q3 to the experience score gives a marginally better statistical fit, but the model without Q3 is preferable because it avoids circularity and still shows the same key finding -- that higher educational level predicts a better patient -- experience score.

**Table 5:** Regression model.

Model	R-square	F	P-value
Experience score (with Q3)	0.056	22.938	0.001
Experience score (without Q3)	0.046	18.987	0.001

Table 6 shows that the inclusion of Q3 slightly increases the effect of educational level on the average score (B = 0.032 vs. 0.030) and results in a slightly higher t-value, suggesting a marginally stronger relationship when Q3 is included. However, in both models, the educational level remains a statistically significant predictor (p = 0.001).

**Table 6:** Model-average score.

Predictor	B (with Q3)	Std. error (with Q3)	t (with Q3)	P-value (with Q3)	B (without Q3)	Std. error (without Q3)	t (without Q3)	P-value (without Q3)
Constant	2.147	0.028	77.721	0.001	2.219	0.028	78.523	0.001
Educational level	0.032	0.007	4.789	0.001	0.030	0.007	4.357	0.001

## Discussion

This study aimed to evaluate the patient experience of individuals with diabetes attending primary healthcare centers in Al-Ahsa, Saudi Arabia. We hypothesized that measuring patient experience would enhance value-based care for patients with diabetes.

This study investigated the experiences of 392 patients with diabetes receiving care at Al-Ahsa Primary Healthcare Centers. Participants ranged in age from 22 to 91 years, with a mean age of 54 years and a nearly equal sex distribution (51.3% male, 48.7% female). Type 2 diabetes was the most predominant type, affecting 61% of the sample. Educational background was diverse: 14% of participants were illiterate, while 25.5% held a bachelor’s degree). 

Overall, participants reported exceptionally positive encounters with primary healthcare services. The mean Likert score was 2.7 out of 3.0, indicating a collective perception of "excellent" satisfaction.

Similarly, other studies in Saudi Arabia have reported high satisfaction rates among patients with chronic conditions in different regions. A cross-sectional analysis by Al Shahrani et al. in Riyadh revealed that approximately 60% of patients with diabetes expressed high satisfaction with their care [28]. Likewise, Al Anzi et al. found a comparable satisfaction level of approximately 65% in their evaluation of patients attending primary health centers in Arar [31].

Collectively, these consistent findings from different regions and patient populations suggest a generally positive experience of primary health care quality across Saudi Arabia.

Certain domains of patient experience were rated highly. "Respect for privacy" received the highest average score (2.93), followed by "time spent during consultation" (2.86) and "nurse attentiveness" (2.84).

In contrast, research outside Saudi Arabia reveals more critical perspectives on primary care experiences. Studies by Abdulhadi et al. in Oman and Falayi et al. in Nigeria showed that patients are generally dissatisfied with the long waiting time, which can last up to four hours [32,33]. In addition, Abdulhadi et al. found that patients were dissatisfied with the way in which they were greeted by healthcare providers, interruption of privacy, poor attention, and eye contact during encounters [32].

Similarly, doctor-patient communication was positively evaluated, with high scores for items such as "doctor's concern for patient questions" (2.88) and "clarity of explanation" (2.80). These consistently favorable ratings suggest strong performance in key interpersonal aspects of care. Such outcomes may reflect the impact of structured communication training provided to healthcare professionals as well as increased institutional emphasis on patient rights and satisfaction metrics within primary care settings.

In contrast to the positive evaluations observed in our study, research from Oman and Pakistan highlights significant challenges in doctor-patient communication. Abdulhadi et al. in Muscat, Oman, mentioned that patients reported a lack of motivation from doctors for asking questions and explaining information regarding laboratory investigations, medications, self-monitoring of blood glucose, and management of hypoglycemia [32]. Similarly, Jalil et al., in Pakistan, found that none of the patients knew which type of diabetes they had, indicating a critical gap in essential disease knowledge [34].

Subgroup analysis revealed statistically significant differences in the patient experience scores based on sex. Male participants reported higher satisfaction levels than female participants (p = 0.02), which may be attributable to differing communication preferences or expectations. In contrast, a study conducted by Jalil et al. in Pakistan found that female patients exhibited higher satisfaction levels with doctor-patient interactions, suggesting that sex may influence patient perceptions and experiences differently across cultural contexts [34]. Higher education levels were also significantly associated with more positive experiences (p = 0.001), suggesting a potential influence of health literacy on patient perception. Supporting this finding, A study conducted in Jeddah by Alsubahi et al. identified a positive correlation between higher educational attainment and patient satisfaction [35]. Conversely, research by Jalil et al. in Pakistan showed that satisfaction levels were higher in patients with lower levels of education, indicating that cultural and healthcare system differences may influence these associations [34].

Occupational status further influenced experience; government employees reported the highest mean experience score (mean = 2.37), while freelance workers reported the lowest (mean = 2.14). This disparity may be attributed to better access to healthcare services among government employees, including comprehensive health insurance and continuity of care, which could contribute to more favorable experiences.

In contrast, a study conducted by Jalil et al. in Pakistan found that unemployed patients reported higher satisfaction levels, suggesting that factors, such as social support or expectations, may play a role in shaping patient perceptions [34]. Similarly, research in Jeddah indicated that patients with high-income jobs reported higher satisfaction levels, possibly because of enhanced access to quality healthcare services [35]. However, studies conducted in Arar, Riyadh, and Abha did not identify any significant associations between patient satisfaction and sociodemographic factors, highlighting the variability of these associations across different settings [28,31,36].

Interestingly, patients with uncontrolled diabetes reported slightly higher satisfaction levels than those with controlled diabetes (p = 0.001), potentially reflecting the increased attention or frequency of care from healthcare providers. Routine follow-up with the care team was also strongly associated with improved experience scores (P = 0.001).

In contrast, Al Anazi et al. found that patients with controlled diabetes had higher satisfaction levels [31]. However, research in Dubai did not find an association between patient satisfaction and diabetes control [37]. Additionally, a study in Nigeria by Falayi et al. concluded that patients with type 1 diabetes experienced higher satisfaction levels (92.9%) than those with type 2 diabetes (77.5%) or those unsure about the type of diabetes (54.8%) [33].

Routine follow-up with the care team was also strongly associated with improved experience scores (P = 0.001). This is proven by some evidence that patient satisfaction with team-based care is better than that with standard care [38].

Finally, linear regression analysis identified educational level as a significant independent predictor of patient experience (B = 0.032, p = 0.001). This finding underscores the role of educational background in shaping patient perceptions of care quality and highlights the importance of targeted communication strategies to enhance understanding, engagement, and satisfaction within primary healthcare settings.

Notably, our study fills an important gap in the literature by exploring the patient experience of diabetes management in primary care settings between those enrolled in team-based care models and those receiving standard care. To the best of our knowledge, no previous study has addressed these comparisons directly. Our findings suggest that integrated, multidisciplinary approaches such as team-based care may play a vital role in enhancing patient experience, particularly by fostering collaboration, continuity, and personalized support.

Taken together, our results underscore the multifactorial nature of patient satisfaction with diabetes care. While some predictors appear consistent across studies, such as the influence of education, others vary according to local healthcare systems and cultural contexts. Further research is warranted to better understand these dynamics and to develop targeted interventions that enhance patient experience across diverse populations. 

 These findings underscore the importance of effective communication, continuity of care, and health literacy in enhancing patient experience. Personalizing care based on educational attainment, gender, and patient expectations may further improve satisfaction. Moreover, subgroup differences highlight the necessity of a patient-centered approach in primary healthcare (PHC) settings, particularly in managing chronic diseases such as diabetes, where individualized support and consistent care delivery can have a substantial impact on the perceived quality of care. 

 This study has several strengths. First, it included a relatively large and diverse sample of diabetic patients recruited from 66 primary healthcare centers (PHCs) across the Al-Ahsa region, which enhances the generalizability of the findings. Second, it addressed both patients with type 1 and type 2 diabetes, offering a comprehensive overview of patient experiences across different disease profiles. 

Additionally, the study employed a broad assessment of patient-centered care by evaluating various aspects of patient experience, including personal safety, laboratory services, and physician-patient interactions. By capturing these multidimensional factors, this study provided a richer understanding of what contributes to patient satisfaction. 

Importantly, this study fills a gap in the literature by being one of the first regional studies to compare satisfaction among patients enrolled in team-based care models versus standard care. 

Despite its contributions, this study had several limitations. The cross-sectional design limits the ability to establish causality between the variables studied. Furthermore, data collection was conducted through telephone interviews, which may have introduced a selection bias. Individuals who lacked access to a phone, were unavailable during call times, or had communication difficulties were excluded, potentially underrepresenting certain subgroups.

The results of this study provide a strong foundation for future investigations aimed at improving the patient experience in PHC settings for individuals with diabetes. Future research should further explore the impact of team-based care models on patient satisfaction, ideally through longitudinal or interventional studies. Moreover, qualitative studies may provide deeper insight into patient expectations, preferences, and barriers to satisfaction, enabling more targeted improvements in service delivery.

## Conclusions

This study provides critical insights into the lived experiences of patients with diabetes receiving care in PHCs across the Al-Ahsa region. Patients reported excellent levels of satisfaction across multiple dimensions of care, particularly in areas related to interpersonal communication, registration efficiency, and privacy. Key predictors of positive patient experiences included higher educational level, consistent follow-up with care teams, and employment in the government sector. Importantly, this study underscores the value of integrating structured team-based care approaches to support improved patient outcomes. The regression model confirmed that educational level is a robust independent predictor of satisfaction, suggesting the need to strengthen health education and communication strategies within PHCs. While the inclusion of Q3 in the experience score slightly improved the statistical fit, the model without Q3 also reliably identified educational level as a key driver of patient experience.

Given the growing burden of diabetes in Saudi Arabia, these findings support the ongoing expansion of primary care services and the importance of patient-centered reforms. Future research should explore longitudinal and interventional designs to evaluate the impact of team-based care and identify actionable strategies to improve healthcare delivery, particularly in vulnerable or underrepresented populations.

## Appendices

**Table 7:** Questionnaire: Demographic data and diabetes history part.

Category	Variable/question	Response options
Clinical data	Most recent A1C level (from medical record)	Numeric value (e.g., 7.2%)
Demographics	Gender	Female/Male
Demographics	Marital status	Married/Separated/Single/Widowed
Demographics	Occupation	Freelance/Government sector/Private sector/Not working/Retired
Demographics	Educational level	Illiterate/Elementary/Secondary school/High school/Diploma/Bachelor
Demographics	Do you have health insurance?	Yes/No/I had it previously
Healthcare access	Usual method for diabetes consultation	Appointment booked/My Health app/Call 937/Without appointment
Continuity of care	Do you follow up with your medical team every time?	Yes/No
Diabetes history	Type of diabetes	Type 1/Type 2/I don’t know
Diabetes history	Method of diagnosis	Early screening programs/I had symptoms/Incidentally
Diabetes history	Current treatment	Pills/Injections/Injections and pills/Only a healthy lifestyle
Diabetes history	Do you know the result of your A1C test?	Yes/No/Not sure
Diabetes complications	Do you have any of the following complications?	Retinopathy/Peripheral neuropathy/Kidney failure/Diabetic foot/None
Comorbidities	Do you have any conditions associated with diabetes?	High blood pressure/High cholesterol/Obesity/None

**Table 8:** Questionnaire: Patient experience part (three-point Likert scale).

Category	Question	Response options
Registration	Ease of appointment booking	Excellent/Average/Poor
Registration	Availability of appointments	Excellent/Average/Poor
Registration	Ease of registration procedures at reception	Excellent/Average/Poor
During Visit	Clarity of navigation plan after registration	Excellent/Average/Poor
During Visit	Waiting time before entering the vital signs area	Excellent/Average/Poor
During Visit	Waiting time before seeing the doctor	Excellent/Average/Poor
During Visit	Time spent during the consultation	Excellent/Average/Poor
Doctor	Explanation provided about your condition	Excellent/Average/Poor
Doctor	Concern shown for your questions	Excellent/Average/Poor
Doctor	Effort to involve you in treatment decisions	Excellent/Average/Poor
Doctor	Discussion of treatment options	Excellent/Average/Poor
Nursing	Nurse’s listening skills	Excellent/Average/Poor
Nursing	Concern nurse showed for your issue	Excellent/Average/Poor
Laboratory	Waiting time before blood sample taken	Excellent/Average/Poor
Laboratory	Time required for lab results	Excellent/Average/Poor
Pharmacy	Availability of prescribed medications	Excellent/Average/Poor
Follow-up	You are given a follow-up appointment after each visit	Excellent/Average/Poor
Personal Matters	Respect for your privacy	Excellent/Average/Poor
Personal Matters	Medical staff ensured your safety (e.g., hand sanitizing)	Excellent/Average/Poor
